# Changes in the anatomical positions of the femoral nerve and artery in the lateral and supine positions: a multicenter retrospective study

**DOI:** 10.1007/s00402-025-05968-9

**Published:** 2025-07-15

**Authors:** Ryuichiro Okuda, Tomonori Tetsunaga, Kazuki Yamada, Tomoko Tetsunaga, Takashi Koura, Tomohiro Inoue, Yasutaka Masada, Tetsuya Yamamoto, Shin Matsumoto, Hisanori Ikuma, Tadashi Komatsubara, Yuki Okazaki, Toshifumi Ozaki

**Affiliations:** 1 Department of Orthopaedic Surgery, Dentistry and Pharmaceutical Sciences, Okayama University Graduate School of Medicine, Okayama, Japan; 2 Department of Musculoskeletal Health Promotion, Faculty of Medicine, Dentistry and Pharmaceutical Sciences, Okayama University, Okayama, Japan; 3 Department of Medical Materials for Musculoskeletal Reconstruction, Faculty of Medicine, Dentistry and Pharmaceutical Sciences, Okayama University, Okayama, Japan; 4 Department of Sports Medicine, Faculty of Medicine, Dentistry and Pharmaceutical Sciences, Okayama University, Okayama, Japan; 5 Department of Orthopaedic Surgery, Kagawa Prefectural Central Hospital, Kagawa, Japan; 6 Department of Orthopaedic Surgery, Okayama Rosai Hospital, Okayama, Japan; 7 Department of Orthopaedic Surgery, Kochi Health Sciences Center, Kochi, Japan; 8 Center for education in medicine and health sciences, Okayama University, Okayama, Japan; 9 Department of Orthopaedic Surgery, Faculty of Medicine, Dentistry and Pharmaceutical Sciences, Okayama University, Okayama, Japan

**Keywords:** Total hip arthroplasty, Femoral artery, Femoral nerve, Computed tomography, Lateral position, Supine position

## Abstract

**Introduction:**

Femoral nerve palsy and femoral artery injury are serious complications of total hip arthroplasty. However, few studies have compared the anatomical positions of these structures in different patient positions. This study aimed to compare the anatomical positions of the femoral nerve and artery in the lateral and supine positions.

**Materials and methods:**

This multicenter retrospective study included 111 patients who underwent lateral and supine computed tomography (CT) from 2016 to 2023. CT images were reconstructed in the anterior pelvic plane. The horizontal distance from the anterior margin of the acetabulum to the femoral nerve (Distance N) and femoral artery (Distance A) was measured. The difference in Distance N between the two positions (ΔLateral–supine Distance N) was calculated by subtracting the supine value from the lateral value.

**Results:**

The average Distance N was 26.5 ± 5.1 mm in the lateral position and 21.1 ± 4.4 mm in the supine position, with the nerve located significantly closer to the acetabulum in the supine position (P < 0.001). Similarly, the average Distance A was 26.8 ± 5.4 mm in the lateral position and 20.4 ± 4.9 mm in the supine position (P < 0.001). Multiple regression analysis showed that Distance N in the lateral position was significantly shorter in female patients and those with low body weight. In addition, low body weight correlated with a smaller ΔLateral–supine Distance N.

**Conclusions:**

The femoral nerve and artery are located closer to the anterior margin of the acetabulum in the supine position than in the lateral position. Low body weight was an independent predictor of shorter Distance N in both positions and a smaller ΔLateral–supine Distance N. These findings underscore the importance of considering patient positioning during total hip arthroplasty, particularly in patients with low body weight, to reduce neurovascular risks.

**Supplementary Information:**

The online version contains supplementary material available at 10.1007/s00402-025-05968-9.

## Introduction

Total hip arthroplasty (THA) is considered the most effective surgical intervention for improving health-related quality of life [1, 2]. Nevertheless, the consequences of adverse events that systematically occur during THA are significant; therefore, it is important to develop protocols that minimize the occurrence of these events [3].

Femoral nerve palsy and femoral artery injury are serious complications of THA. Previous studies have reported varying incidence rates of femoral nerve palsy according to the surgical approach [4–6]. Vascular injury in THA, with a reported incidence rate ranging from 0.1 to 0.2%, is less common than neurological injury but is more emergent [7]. In THA, the most common identifiable cause of intraoperative nerve injury is compression by retractors [3], with the external iliac and femoral arteries being the most frequently injured vascular structures [7]. The primary etiology of femoral nerve injury is retractor placement along the anterior acetabular rim [8–10]; in particular, acetabular retractors placed too far medially are the most frequent causes of femoral artery injury [11, 12].

Understanding the anatomical variations and positional changes in these structures is crucial for minimizing these risks. Although previous reports have underscored the importance of patient positioning during surgery, comprehensive analyses comparing the lateral and supine positions are limited [13]. Therefore, the current study aimed to compare the anatomical positions of the femoral nerve and artery in the lateral and supine positions.

## Materials and methods

### Study design

This multicenter retrospective study was conducted at three institutes in accordance with the principles of the Declaration of Helsinki. The study protocol was approved by the institutional ethics committee (registration number: 2310-016). Informed consent was obtained in the form of opt-out on the website.

Medical records of 151 patients who underwent computed tomography (CT) in the lateral and supine positions for preoperative spine-surgery planning were reviewed. Patients who underwent lateral and supine CT scans from April 2016 to March 2023 were included in the study. Although the scans were performed on different days, both were completed within 1 month of each other. Cases in which surgery was performed between the two scan dates were excluded. Supine CT scans were obtained for the diagnosis of spinal disorders, whereas lateral CT scans were conducted for preoperative evaluation before lateral lumbar interbody fusion (LLIF), specifically to assess the positions of critical anatomical structures—such as the aorta, inferior vena cava, kidneys, ureters, and intestines—in the lateral decubitus position. Lateral CT scans were performed in a standardized true lateral decubitus position, with the hip slightly flexed and the pelvis perpendicular to the CT table, using positioning cushions. Cases in which lateral CT could not be performed in the designated position due to severe pain were also excluded. Forty patients were excluded due to an inadequate CT scan range (*n* = 21), severe hip deformity (*n* = 9), a history of hip surgery (*n* = 7), or a history of pelvic fracture (*n* = 3). Ultimately, 111 patients were included in this analysis (Table 1).

**Table 1:** Patient demographics

	Total (*n* = 111)
Age (years)	68.3 ± 10.5
Sex (female / male)	72 / 39
Body height (cm)	157.7 ± 9.4
Body weight (kg)	58.3 ± 11.4
Body mass index (kg/m^2^)	23.2 ± 4.1
Diagnosis (D/ Tr / Tu / I)	87 / 15 / 5 / 4

Data presented as mean ± SD

*D* degenerative disease; *Tr* trauma; *Tu* tumor; *I* infection

**Fig. 1:**
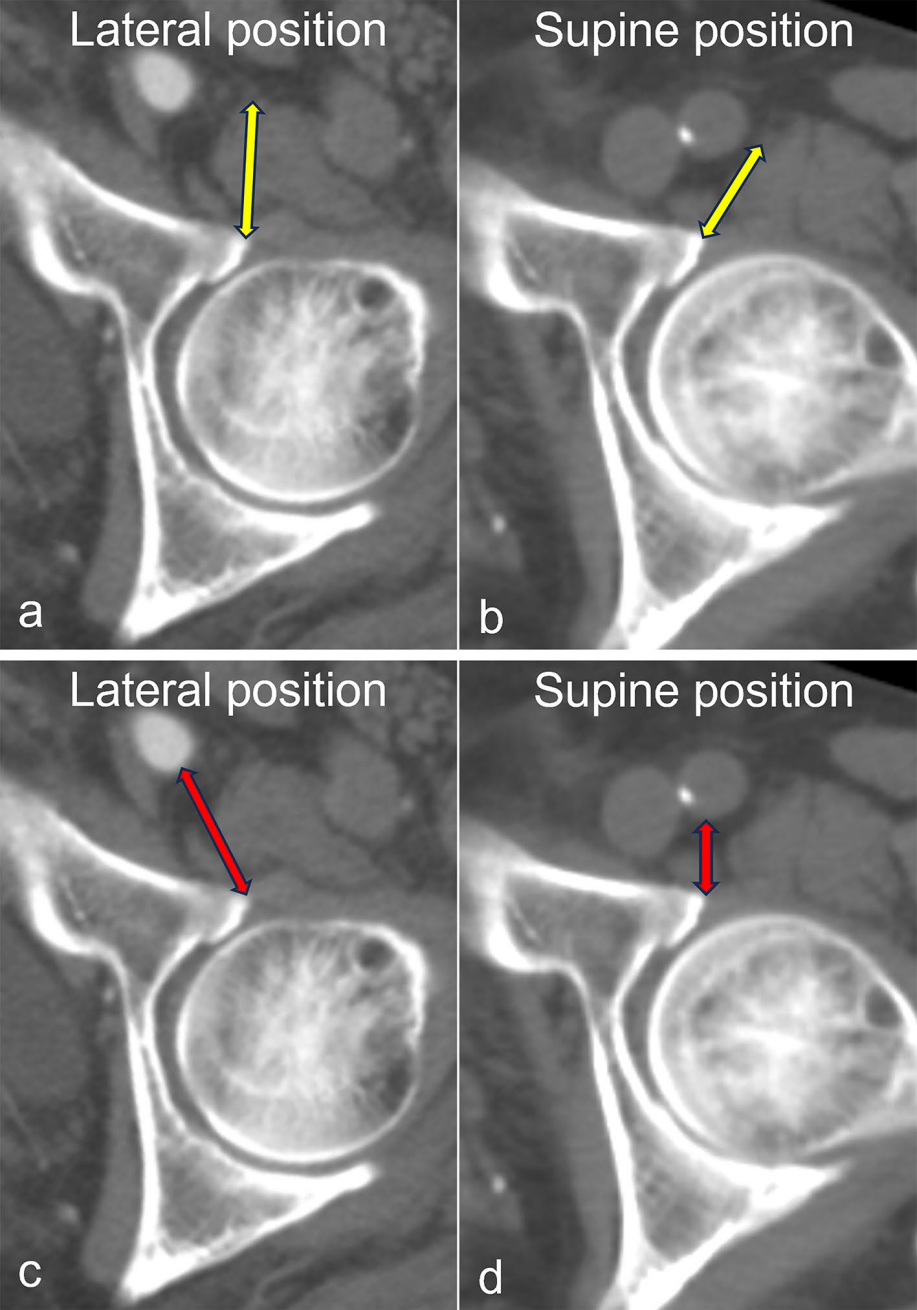
Computed tomography images showing Distances N and A. (**a**) Distance N in the lateral position. (**b**) Distance N in the supine position. (**c**) Distance A in the lateral position. (**d**) Distance A in the supine position. Distance N, distance from the anterior margin of the acetabulum to the femoral nerve; Distance A, distance from the anterior margin of the acetabulum to the femoral artery

The lateral and supine CT images were reconstructed using the anatomical pelvic plane coordinate system. The distance from the anterior margin of the acetabulum to the femoral nerve (Distance N) and femoral artery (Distance A) in a horizontal section slice, including the center of the femoral head, was measured. ΔLateral–supine Distance N was defined as the value obtained by subtracting Distance N in the supine position from Distance N in the lateral position. Similarly, Δlateral–supine Distance A was defined as the value obtained by subtracting Distance A in the supine position from Distance A in the lateral position. Distances N and A were measured using a digital caliper tool on SYNAPSE VINCENT (Fujifilm, Japan; Fig. 1).

Two blinded board-certified orthopedic surgeons served as the raters. Each rater performed all measurements independently. The measurement of these distances was first standardized with 20 training cases by the two raters prior to the evaluation of the 111 study cases. The two distances were measured on CT by comparing the lateral and supine positions.

### Statistical analysis

The anatomical positions of the femoral nerve and artery in the lateral and supine positions were compared using a paired *t*-test. All patients were divided into two groups according to the mean values of Distances N and A in the lateral and supine positions. A univariate analysis was conducted to compare age, sex, body height, body weight, and diagnosis between the groups. Normally distributed variables were compared using Student’s *t*-test, whereas categorical variables were analyzed using the chi-squared or Fisher's exact test. Factors predictive of Distance N or A were identified via a multiple linear regression analysis. Potential predictive variables were included in the multivariate model if *P* values of < 0.05 were obtained on the univariate analysis.

The patients were also divided into two groups according to the amount of Δlateral–supine Distances N and A. Subsequently, a univariate analysis was conducted to compare age, sex, body height, body weight, and diagnosis between the groups. Normally distributed variables were compared using Student’s *t*-test, whereas categorical variables were analyzed using the chi-squared or Fisher's exact test. Factors predictive of Δlateral–supine Distance N or A were identified via a multiple linear regression analysis. Potential predictive variables were included in the multivariate model if *P* values of < 0.1 were obtained on the univariate analysis.

Intra- and inter-observer reliability for the measurement of each distance were assessed using intraclass correlation coefficients (ICCs). Two orthopedic surgeons independently performed all measurements. Intra-observer reliability was evaluated with repeated measurements taken after a 2-week interval. Correlations between body height and distance were also evaluated. Statistical analyses were performed using EZR software (Saitama Medical Center, Jichi Medical University, Saitama, Japan) [14], with statistical significance set at *P* < 0.05.

## Results

A total of 111 patients were analyzed, as shown in Table 1. The average patient age was 68.3 ± 10.5 years, and the cohort comprised 72 women and 39 men. The average body height was 157.7 ± 9.4 cm, whereas the average body weight was 58.3 ± 11.4 kg. The mean body mass index was 23.2 ± 4.1 kg/m^2^. With respect to diagnosis, 87 patients had degenerative diseases, 15 had trauma, 5 had tumors, and 4 had infections.

Distance N was significantly shorter in the supine position than in the lateral position (*P* < 0.001; Table 2). Similarly, Distance A was significantly shorter in the supine position than in the lateral position (*P* < 0.001).

**Table 2:** CT parameters in the lateral and supine positions

	Lateral position	Supine position	*P* value
Distance N (mm)	26.5 ± 5.1	21.1 ± 4.4	< 0.001
Distance A (mm)	26.8 ± 5.4	20.4 ± 4.9	< 0.001

Data presented as mean ± SD

*CT* computed tomography; *Distance N* distance from the anterior margin of the acetabulum to the femoral nerve; *Distance A* distance from the anterior margin of the acetabulum to the femoral artery

Patient characteristics were examined in relation to Distances N and A in the lateral and supine positions using the univariate analysis (Table 3). The correlation analysis between patient characteristics and Distances N and A in the lateral and supine positions is provided in the supplementary information (Supplementary Fig. 1–5). Patients were divided into two groups according to the average Distance N or A. Regarding Distance N in the lateral position, significant differences in sex (*P* = 0.010), height (*P* < 0.001), and body weight (*P* < 0.001) were observed. As for Distance A in the lateral position, sex (*P* < 0.001), height (*P* < 0.001), and body weight (*P* < 0.001) showed significant differences. With respect to Distance N in the supine position, there were significant differences in height (*P* < 0.001) and body weight (*P* < 0.001). As for Distance A in the supine position, significant differences in sex (*P* < 0.001), height (*P* < 0.001), and body weight (*P* < 0.001) were noted.

**Table 3:** Univariate analysis of Distances N and A in the lateral and supine positions

	Distance N in the lateral position		
	> 26.5 mm (*n* = 52)	≤ 26.5 mm (*n* = 59)	*P* value
Age (years)	67.2 ± 10.2	69.5 ± 10.8	0.256
Sex (female / male)	22 / 30	50 / 9	0.010
Body height (cm)	160.9 ± 9.6	152.8 ± 7.0	< 0.001
Body weight (kg)	62.2 ± 11.2	52.0 ± 9.7	< 0.001
Diagnosis (D/ Tr / Tu / I)	44 / 4 / 3 / 1	43 / 11 / 2 / 3	0.103

	Distance A in the lateral position		
	> 26.8 mm (*n* = 59)	≤ 26.8 mm (*n* = 52)	*P* value
Age (years)	66.3 ± 11.0	70.0 ± 9.8	0.067
Sex (female / male)	27 / 32	45 / 7	< 0.001
Body height (cm)	161.5 ± 9.2	153.1 ± 7.6	< 0.001
Body weight (kg)	62.7 ± 11.2	52.8 ± 9.9	< 0.001
Diagnosis (D/ Tr / Tu / I)	48 / 7 / 2 / 2	39 / 8 / 3 / 2	0.972

	Distance N in the supine position		
	> 21.1 mm (*n* = 49)	≤ 21.1 mm (*n* = 62)	*P* value
Age (years)	67.8 ± 9.2	68.6 ± 11.4	0.712
Sex (female / male)	23 / 26	49 / 13	0.069
Body height (cm)	160.2 ± 9.9	154.8 ± 8.3	0.002
Body weight (kg)	61.8 ± 11.8	54.3 ± 10.6	< 0.001
Diagnosis (D/ Tr / Tu / I)	40 / 5 / 2 / 2	47 / 10 / 3 / 2	0.272

	Distance A in the supine position		
	> 20.4 mm (*n* = 46)	≤ 20.4 mm (*n* = 65)	*P* value
Age (years)	66.3 ± 10.0	69.9 ± 10.7	0.074
Sex (female / male)	25 / 21	47 / 18	< 0.001
Body height (cm)	160.9 ± 9.4	154.0 ± 8.2	< 0.001
Body weight (kg)	63.3 ± 10.9	52.8 ± 10.1	< 0.001
Diagnosis (D/ Tr / Tu / I)	39 / 5 / 2 / 0	48 / 10 / 3 / 4	0.394

Data presented as mean ± SD

*D* degenerative disease; *Tr* trauma; *Tu* tumor; *I* infection

Patient characteristics were also analyzed in relation to Distances N and A in the lateral and supine positions via a multiple linear regression analysis (Table 4). The factors determining short Distance N in the lateral position were female sex (*P* = 0.010) and low body weight (*P* = 0.003), whereas the factor determining short Distance A in the lateral position was female sex (*P* = 0.002) only. The factor determining short Distances N and A in the supine position was low body weight (*P* = 0.002 and *P* = 0.044, respectively).

**Table 4:** Multiple linear regression analysis of Distances N and A in the lateral and supine positions

			95% CI		
Variables	Partial regression coefficient	Standard error	Lower	Upper	*P* value
Distance N in the lateral position					
Sex (female)	– 0.290	0.111	– 0.510	– 0.070	0.010
Body height	0.006	0.006	– 0.006	0.018	0.340
Body weight	0.012	0.004	0.004	0.020	0.003
Distance A in the lateral position					
Sex (female)	– 0.176	0.112	– 0.399	0.047	0.002
Body height	0.010	0.006	– 0.003	0.022	0.122
Body weight	0.013	0.004	0.005	0.021	0.121
Distance N in the supine position					
Body height	0.007	0.006	– 0.004	0.018	0.202
Body weight	0.014	0.004	0.005	0.022	0.002
Distance A in the supine position					
Sex (female)	– 0.003	0.123	– 0.247	0.241	0.978
Body height	0.009	0.007	– 0.004	0.023	0.182
Body weight	0.009	0.005	0.001	0.018	0.044

*CI*, confidence interval

The average Δlateral–supine Distance N was 5.4 ± 3.0 mm, and the average Δlateral–supine Distance A was 6.5 ± 4.7 mm. Patient characteristics were examined in relation to Δlateral–supine Distances N and A via the univariate analysis (Table 5). Patients were divided into two groups according to the average Δlateral–supine Distance N or A. With respect to Δlateral–supine Distance N, significant differences in body weight were observed (*P* = 0.007); however, no significant differences in age, sex, body height, and diagnosis were found. Regarding Δlateral–supine Distance A, significant differences in sex (*P* = 0.010) and body height (*P* = 0.048) were observed; however, there were no significant differences in age, sex, body height, and diagnosis.

**Table 5:** Univariate analysis of “Δlateral–supine Distance N” and “Δlateral–supine Distance A”

	ΔLateral–supine Distance N		
	> 5.4 mm (*n* = 61)	≤ 5.4 mm (*n* = 50)	*P* value
Age (years)	67.3 ± 10.7	69.4 ± 10.3	0.298
Sex (female / male)	36 / 25	36 / 14	0.168
Body height (cm)	158.5 ± 9.0	155.2 ± 9.5	0.065
Body weight (kg)	60.1 ± 11.2	54.2 ± 11.4	0.007
Diagnosis (D/ Tr / Tu / I)	49 / 5 / 4 / 3	38 / 10 / 1 / 1	0.216

	ΔLateral–supine Distance A		
	> 6.5 mm (*n* = 55)	≤ 6.5 mm (*n* = 56)	*P* value
Age (years)	67.7 ± 12.1	68.9 ± 8.8	0.555
Sex (female / male)	29 / 26	43 / 13	0.010
Body height (cm)	158.8 ± 9.8	155.3 ± 8.6	0.048
Body weight (kg)	58.6 ± 12.2	56.3 ± 11.1	0.308
Diagnosis (D/ Tr / Tu / I)	39 / 10 / 3 / 3	48 / 5 / 2 / 1	0.295

Data presented as mean ± SD

*D* degenerative disease; *Tr* trauma; *Tu* tumor; *I* infection

Patient characteristics were also analyzed in relation to Δlateral–supine Distances N and A using the multiple linear regression analysis (Table 6). A smaller Δlateral–supine Distance N was observed in patients with low body weight (*P* = 0.045). As for Δlateral–supine Distance A, no significant differences in sex and body height were found.

**Table 6:** Multiple linear regression analysis of “Δlateral–supine Distance N” and “Δlateral–supine Distance A”

ΔLateral–supine Distance N					
			95% CI		
Variables	Partial regression coefficient	Standard error	Lower	Upper	*P* value
Body height	0.003	0.006	– 0.008	0.015	0.591
Body weight	0.010	0.005	– 0.001	0.019	0.045

ΔLateral–supine Distance A					
			95% CI		
Variables	Partial regression coefficient	Standard error	Lower	Upper	*P* value
Sex (female)	– 0.236	0.129	– 0.491	– 0.019	0.069
Body height	– 0.002	0.007	– 0.011	0.015	0.740

Data presented as mean ± SD

*CI* confidence interval

The ICCs for Distance N in the lateral and supine positions indicated good intra- and inter-observer reliability, whereas the ICCs for Distance A in the lateral and supine positions suggested excellent intra- and inter-observer reliability (Table 7).

**Table 7:** Intra- and inter-observer reliability

Reliability	Distance N	Distance A
Intra-observer reliability in the lateral position	0.84	0.98
Inter-observer reliability in the lateral position	0.84	0.92
Intra-observer reliability in the supine position	0.86	0.96
Inter-observer reliability in the supine position	0.82	0.92

*Distance N* distance from the anterior margin of the acetabulum to the femoral nerve; *Distance A* distance from the anterior margin of the acetabulum to the femoral artery

## Discussion

The current study revealed that Distances N and A were significantly shorter in the supine position than in the lateral position and that low body weight was an independent predictor of shorter Distance N in the lateral and supine positions, as well as a smaller Δlateral–supine Distance N.

The distance between the femoral nerve and acetabular rim has been shown by previous studies to be significantly shorter in the supine position than in the lateral position [13]. This anatomical variation is crucial because it suggests a higher risk of femoral nerve injury in the supine position during THA. Notably, our study reinforces this finding by demonstrating that Distance N (femoral nerve) and Distance A (femoral artery) were significantly shorter in the supine position.

Femoral nerve palsy after THA can lead to quadriceps muscle weakness and abnormal sensation in the thigh, negatively affect postoperative rehabilitation, and reduce patient satisfaction [15, 16]. The incidence of femoral nerve palsy is the highest in anterolateral THA (0.64% of 17,350 primary THA cases), followed by anterior (0.4%), posterior (0.045%), and lateral THAs (0.026%) [4]. Therefore, particular attention should be paid to femoral nerve palsy during supine THA compared to other approaches performed in the lateral decubitus position.

Previous reports indicated that the incidence of femoral nerve palsy in THA using the direct anterior approach was 1.1% (3 out of 273 cases) [17]. Femoral nerve palsy was attributed to the surgeon's learning curve because it occurred only in the first 20 cases performed by the surgeon and did not recur thereafter; the insertion of the retractor into the anterior wall of the acetabulum was also identified as a cause of femoral nerve palsy. Furthermore, an anatomical study of the femoral nerve course in 84 limbs showed that the nerve was closest to the acetabular rim at 90° anteriorly from a line drawn from the anterior superior iliac spine to the center of the acetabulum (0°: 33.2 mm, 30°: 24.4 mm, 60°: 18.4 mm, 90°: 16.6 mm, 120°: 17.9 mm, 150°: 23.2 mm) [18]. In the current study, we also examined the point closest to the femoral nerve and acetabular rim at the center of the femoral head. Distance N in the lateral position was longer than that in the supine position. However, Δlateral–supine Distance N in patients with low body weight was small. Therefore, care must be taken to avoid femoral nerve injury even in the lateral position.

As shown by previous studies, the nerve potential decreases when a retractor is placed anterior to the acetabulum [19, 20]. This decrease in nerve potential is directly related to the mechanical compression caused by the retractor, which can lead to temporary or permanent nerve damage. Additionally, a documented correlation exists between the iliopsoas muscle thickness and distance from the femoral nerve [18]. Our findings further support this correlation, suggesting that patients with thinner iliopsoas muscles may be at an increased risk for nerve injury during THA because of the reduced protective space between the nerve and surgical instruments.

The anatomical positions of the femoral nerve and artery significantly varied between the lateral and supine positions, highlighting the importance of patient positioning and individual anatomical variations during surgical planning. For instance, preoperative planning should include imaging studies to assess the relative positions of critical neurovascular structures in different positions, and intraoperative nerve monitoring may be performed to minimize the risk of injury in patients with short Distance N or small Δlateral–supine Distance N.

Our study had some limitations. The use of CT imaging, which has a lower ICC than magnetic resonance imaging (MRI) [13], might have affected the accuracy. CT scans provide detailed bone anatomy but may not capture soft tissue structures as accurately as MRI, potentially leading to less precise measurements. Additionally, lateral CT scans were performed with the hip slightly flexed, which might have influenced the measurements [21]. This flexed position can alter the spatial relationship between the acetabulum and neurovascular structures, potentially affecting the generalizability of our results. However, surgery is performed in the lateral position and the hip joint is slightly flexed; therefore, we consider that the results of our study are clinically relevant. Another limitation of this study is that its design limits the conclusions to anatomical differences in the distance of the femoral nerve and artery between the lateral and supine positions. It does not provide direct evidence of clinical implications, such as the correlation between patient positioning and the risk of neurovascular injury. Therefore, we cannot offer recommendations regarding the choice of surgical position or approach based on our findings. In clinical practice, careful placement of the anterior retractor is essential, regardless of whether the procedure is performed in the supine or lateral position. Furthermore, this study only included cases of preoperative spinal surgery and did not investigate patients with hip disorders. In hips classified as Crowe group III or IV, the femoral blood vessels pass close to the anterior wall of the acetabulum, requiring caution to avoid vascular injury [22]. Patients undergoing spine surgery may have different anatomical characteristics from those with hip disorders, which could influence the general applicability of our findings to the typical THA population. Future research should focus on a broader demographic group, including those with various hip pathologies, to validate and extend these findings.

As unexpected results, multivariate analysis showed that low body weight was a risk factor for a smaller Δlateral–supine Distance N, whereas no specific risk factors for Δlateral–supine Distance A were identified. No studies have addressed Δlateral–supine Distance N or Δlateral–supine Distance A, indicating a lack of insight into these measures. One possible hypothesis for this result is that the femoral nerve is located medial and anterior to the iliopsoas muscle, and the amount of movement due to positional differences may be related to the iliopsoas muscle mass. Iliopsoas muscle volume positively correlates with body weight [23, 24], and the psoas muscle index is a useful predictor of THA outcomes [25]. Patients with higher body weight may have greater iliopsoas muscle volume, potentially resulting in larger Δlateral-supine Distance N. By contrast, the femoral artery is located medial to the femoral nerve, separated by the iliopectineal fascia, and has little mobility, making it less influenced by the iliopsoas muscle volume [26].

## Conclusions

This study revealed that the femoral nerve and artery were located closer to the anterior margin of the acetabulum in the supine position than in the lateral position and that low body weight was an independent predictor of shorter Distance N in the lateral and supine positions, as well as smaller lateral–Supine Distance N. The findings underscore the importance of considering patient positioning during total hip arthroplasty to reduce the risk of femoral nerve and artery complications, particularly in patients with low body weight.

## Supplementary Information

Below is the link to the electronic supplementary material.

Supplementary file1 (DOCX 326 KB)

## Data Availability

No datasets were generated or analysed during the current study.
