# Supplementary material for: Changes in the anatomical positions of the femoral nerve and artery in the lateral and supine positions: a multicenter retrospective study
Source: Arch Orthop Trauma Surg. 2025 Jul 15;145(1):373. doi: 10.1007/s00402-025-05968-9 (PMC12263717; doi:10.1007/s00402-025-05968-9)
Supplement: Supplementary file 1 — Supplementary file1 (DOCX 326 KB) [file 402_2025_5968_MOESM1_ESM.docx]

**Supplementary Information for**

**Changes in the anatomical positions of the femoral nerve and artery in the lateral and supine positions: A multicenter retrospective study**

Ryuichiro Okuda, MD^1^, Tomonori Tetsunaga, MD, PhD^2^*, Kazuki Yamada, MD, PhD^3^, Tomoko Tetsunaga, MD, PhD^4^, Takashi Koura, MD^1^, Tomohiro Inoue, MD^1^, Yasutaka Masada, MD^1^, Tetsuya Yamamoto^1^, Shin Matsumoto^1^, Hisanori Ikuma, MD, PhD^5^, Tadashi Komatsubara, MD^6^, Yuki Okazaki, MD, PhD^1, 7^, Toshifumi Ozaki, MD, PhD^1, 8^

1 Department of Orthopaedic Surgery, Okayama University Graduate School of Medicine, Dentistry and Pharmaceutical Sciences, Okayama, Japan

2 Department of Musculoskeletal Health Promotion, Faculty of Medicine, Dentistry and Pharmaceutical Sciences, Okayama University, Okayama, Japan

3 Department of Medical Materials for Musculoskeletal Reconstruction, Faculty of Medicine, Dentistry and Pharmaceutical Sciences, Okayama University, Okayama, Japan

4 Department of Sports Medicine, Faculty of Medicine, Dentistry and Pharmaceutical Sciences, Okayama University, Okayama, Japan.

5 Department of Orthopaedic Surgery, Kagawa Prefectural Central Hospital, Kagawa, Japan

6 Department of Orthopaedic Surgery, Okayama Rosai Hospital, Okayama, Japan

7 Center for education in medicine and health sciences, Okayama University, Okayama, Japan

8 Department of Orthopaedic Surgery, Faculty of Medicine, Dentistry and Pharmaceutical Sciences, Okayama University, Okayama, Japan

* Corresponding author

Tomonori Tetsunaga, MD, PhD, Department of Musculoskeletal Health Promotion, Faculty of Medicine, Dentistry and Pharmaceutical Sciences, Okayama University, 22-5-1 Shikata-cho, Kitaku, Okayama City, Okayama Prefecture 700-8558, Japan. Tel +81-86-235-7273; E-mail: tomonori_t31@yahoo.co.jp

**This file includes:**

Supplementary Figures 1 to 5


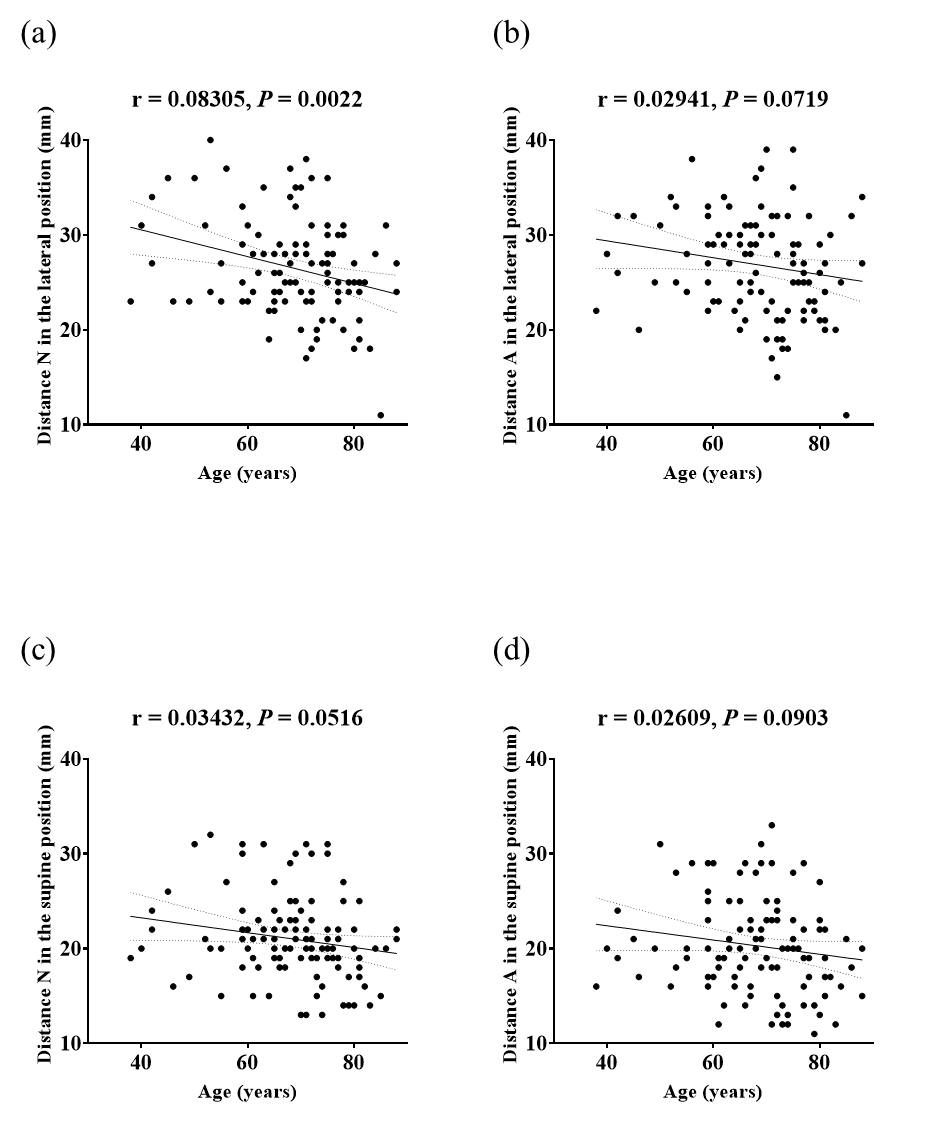


**Supplementary Figure 1. Relationships between age and Distance N and Distance A in lateral and supine positions.**

Scatter plots show the correlation between age and the distance from the anterior margin of the acetabulum to the femoral nerve (Distance N) and femoral artery (Distance A) in the lateral and supine positions. Each data point represents an individual case. The solid line indicates the simple linear regression line (best-fit line), whereas the dashed lines represent the 95% confidence interval for the regression line. (a) Distance N in the lateral position vs. age. (b) Distance A in the lateral position vs. age. (c) Distance N in the supine position vs. age. (d) Distance A in the supine position vs. age.


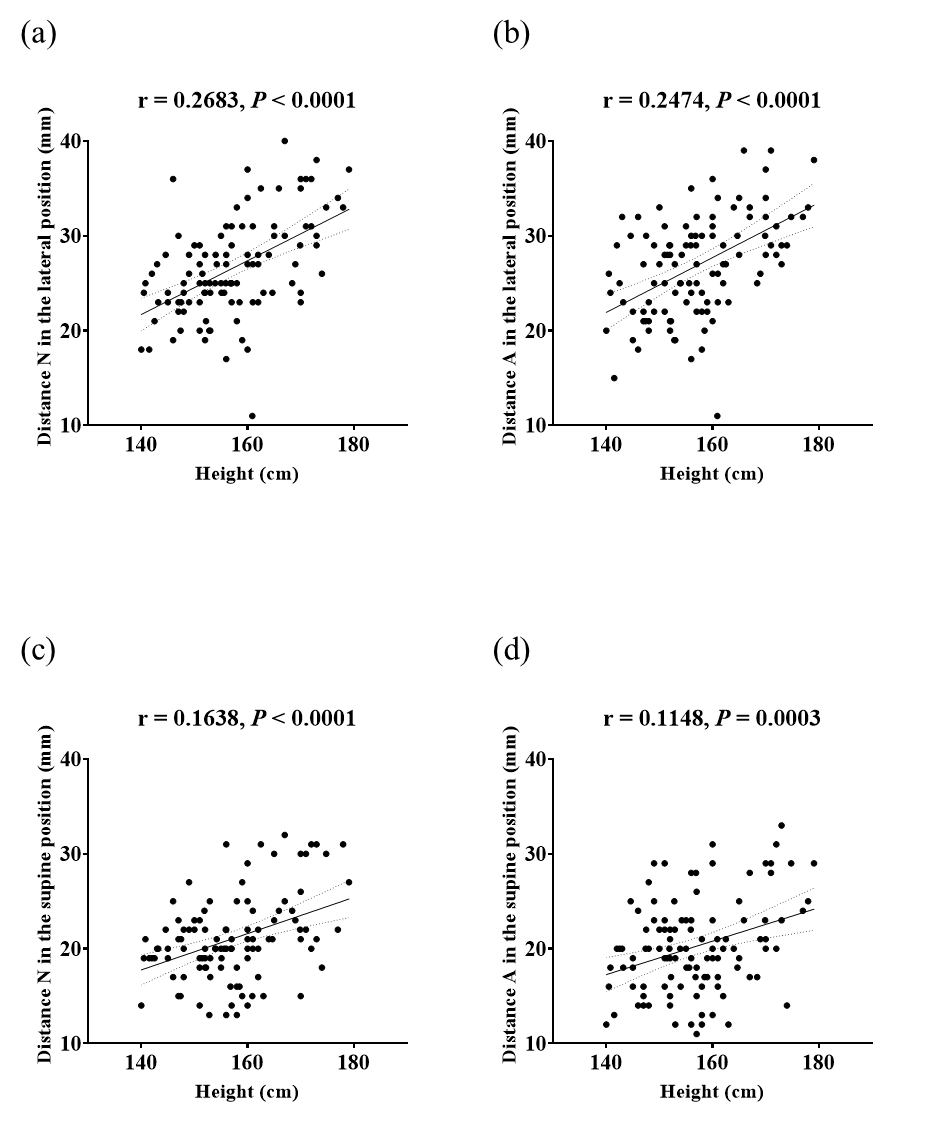
 **Supplementary Figure 2. Relationships between height and Distance N and Distance A in lateral and supine positions.**

Scatter plots display the correlation between height and the distance from the anterior margin of the acetabulum to the femoral nerve (Distance N) and femoral artery (Distance A) in lateral and supine positions. Each data point represents an individual case. The solid line shows the simple linear regression line (best-fit line), whereas the dashed lines indicate the 95% confidence interval for the regression line. (a) Distance N in the lateral position vs. height. (b) Distance A in the lateral position vs. height. (c) Distance N in the supine position vs. height. (d) Distance A in the supine position vs. height.

**
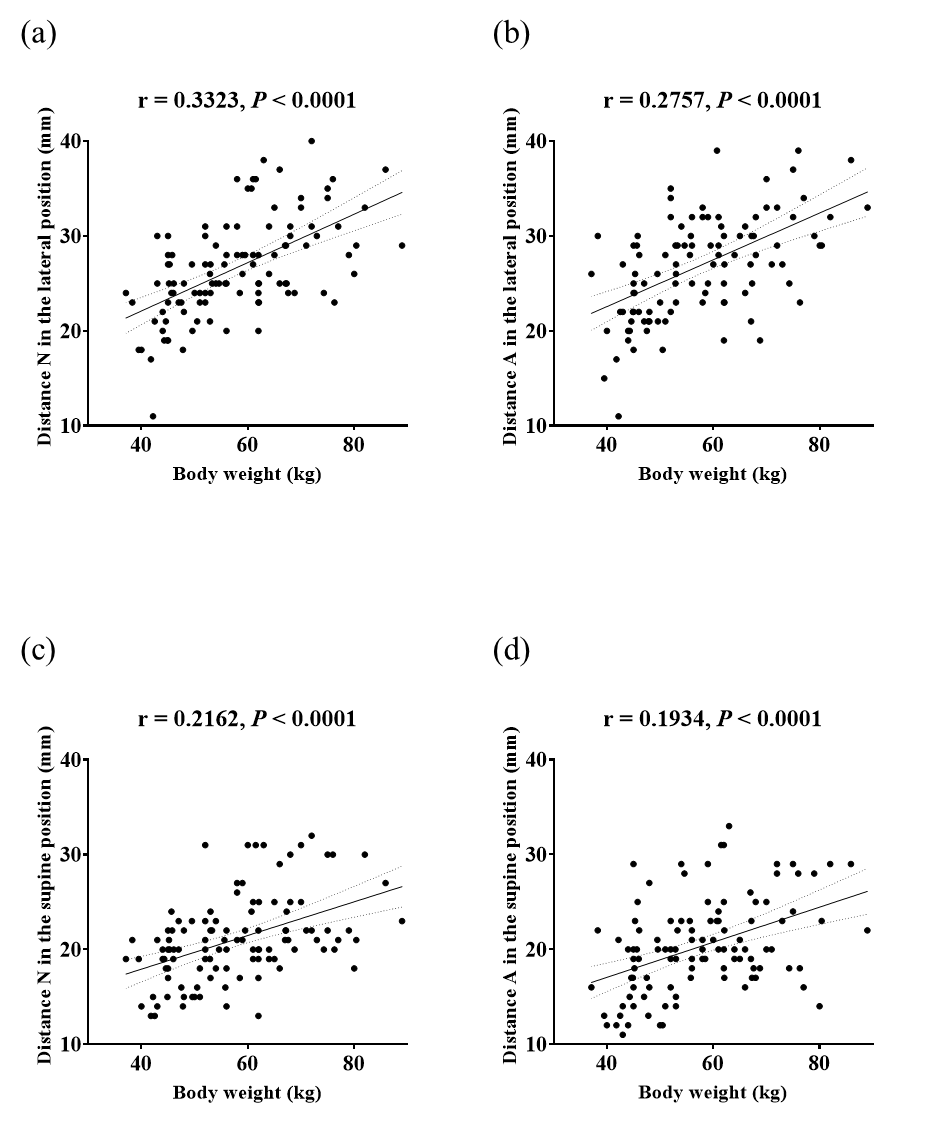
**

**Supplementary Figure 3. Relationships between body weight and Distance N and Distance A in lateral and supine positions.**

Scatter plots display the correlation between body weight and the distance from the anterior margin of the acetabulum to the femoral nerve (Distance N) and femoral artery (Distance A) in lateral and supine positions. Each data point represents an individual case. The solid line represents the simple linear regression line (best-fit line), whereas the dashed lines indicate the 95% confidence interval for the regression line. (a) Distance N in the lateral position vs. body weight. (b) Distance A in the lateral position vs. body weight. (c) Distance N in the supine position vs. body weight. (d) Distance A in the supine position vs. body weight. **Supplementary Figure 4. Comparison of Distance N and Distance A by sex in lateral and supine positions.**

Graphs display the distance from the anterior margin of the acetabulum to the femoral nerve (Distance N) and femoral artery (Distance A) in lateral and supine positions, categorized by sex (female and male). Each dot represents an individual case, with the solid horizontal lines indicating the mean values and 95% confidence intervals. (a) Distance N in the lateral position vs. sex. (b) Distance A in the lateral position vs. sex. (c) Distance N in the supine position vs. sex. (d) Distance A in the supine position vs. sex.

 **Supplementary Figure 5. Comparison of Distance N and Distance A by diagnosis in lateral and supine positions.**

Graphs display the distance from the anterior margin of the acetabulum to the femoral nerve (Distance N) and femoral artery (Distance A) in lateral and supine positions, categorized by diagnosis (D, degenerative disease; Tr, trauma; Tu, tumor; I, infection). Each dot represents an individual case, with the solid horizontal lines indicating the mean values and 95% confidence intervals. (a) Distance N in the lateral position vs. diagnosis. (b) Distance A in the lateral position vs. diagnosis. (c) Distance N in the supine position vs. diagnosis. (d) Distance A in the supine position vs. diagnosis.
